# Sex-specific cardiovascular remodeling leads to a divergent sex-dependent development of heart failure in aged hypertensive rats

**DOI:** 10.1007/s11357-024-01160-w

**Published:** 2024-04-24

**Authors:** Árpád Kovács, Saltanat Zhazykbayeva, Melissa Herwig, Gábor Á. Fülöp, Tamás Csípő, Nikolett Oláh, Roua Hassoun, Heidi Budde, Hersh Osman, Mustafa Kaçmaz, Kornelia Jaquet, Dániel Priksz, Béla Juhász, Ibrahim Akin, Zoltán Papp, Wolfgang E. Schmidt, Andreas Mügge, Ibrahim El-Battrawy, Attila Tóth, Nazha Hamdani

**Affiliations:** 1 Department of Cellular and Translational Physiology, Institute of Physiology, Ruhr University Bochum, 44801 Bochum, Germany; 2 Institut für Forschung und Lehre (IFL), Molecular and Experimental Cardiology, Ruhr University Bochum, 44801 Bochum, Germany; 3 Division of Clinical Physiology, Department of Cardiology, Faculty of Medicine, University of Debrecen, Debrecen, 4032 Hungary; 4 Department of Cardiology, St. Josef-Hospital, UK RUB, Ruhr University Bochum, 44801 Bochum, Germany; 5 HCEMM-SU Cardiovascular Comorbidities Research Group, Department of Pharmacology and Pharmacotherapy, Semmelweis University, Budapest, 1089 Hungary; 6 Department of Pharmacology and Pharmacotherapy, Faculty of Medicine, University of Debrecen, Debrecen, 4032 Hungary; 7 University Medical Centre Mannheim, Medical Faculty Mannheim, Heidelberg University, 68167 Mannheim, Germany; 8 Research Centre for Molecular Medicine, University of Debrecen, Debrecen, 4032 Hungary; 9 Department of Medicine I, St. Josef Hospital, UK RUB, Ruhr University Bochum, 44801 Bochum, Germany; 10 Department of Cardiology and Angiology, Bergmannsheil University Hospitals, UK RUB, Ruhr University of Bochum, 44789 Bochum, Germany; 11 Department of Physiology, Cardiovascular Research Institute, Maastricht University, 6229 ER Maastricht, The Netherlands

**Keywords:** Heart failure with preserved ejection fraction, Sex, PKG, CamKII, Diastolic dysfunction

## Abstract

**Introduction:**

The prevalence of heart failure with preserved ejection fraction (HFpEF) is continuously rising and predominantly affects older women often hypertensive and/or obese or diabetic. Indeed, there is evidence on sex differences in the development of HF. Hence, we studied cardiovascular performance dependent on sex and age as well as pathomechanisms on a cellular and molecular level.

**Methods:**

We studied 15-week- and 1-year-old female and male hypertensive transgenic rats carrying the mouse Ren-2 renin gene (TG) and compared them to wild-type (WT) controls at the same age. We tracked blood pressure and cardiac function via echocardiography. After sacrificing the 1-year survivors we studied vascular smooth muscle and endothelial function. Isolated single skinned cardiomyocytes were used to determine passive stiffness and Ca^2+^-dependent force. In addition, Western blots were applied to analyse the phosphorylation status of sarcomeric regulatory proteins, titin and of protein kinases AMPK, PKG, CaMKII as well as their expression. Protein kinase activity assays were used to measure activities of CaMKII, PKG and angiotensin-converting enzyme (ACE).

**Results:**

TG male rats showed significantly higher mortality at 1 year than females or WT male rats. Left ventricular (LV) ejection fraction was specifically reduced in male, but not in female TG rats, while LV diastolic dysfunction was evident in both TG sexes, but LV hypertrophy, increased LV ACE activity, and reduced AMPK activity as evident from AMPK hypophosphorylation were specific to male rats. Sex differences were also observed in vascular and cardiomyocyte function showing different response to acetylcholine and Ca^2+^-sensitivity of force production, respectively cardiomyocyte functional changes were associated with altered phosphorylation states of cardiac myosin binding protein C and cardiac troponin I phosphorylation in TG males only. Cardiomyocyte passive stiffness was increased in TG animals. On a molecular level titin phosphorylation pattern was altered, though alterations were sex-specific. Thus, also the reduction of PKG expression and activity was more pronounced in TG females. However, cardiomyocyte passive stiffness was restored by PKG and CaMKII treatments in both TG sexes.

**Conclusion:**

Here we demonstrated divergent sex-specific cardiovascular adaptation to the over-activation of the renin-angiotensin system in the rat. Higher mortality of male TG rats in contrast to female TG rats was observed as well as reduced LV systolic function, whereas females mainly developed HFpEF. Though both sexes developed increased myocardial stiffness to which an impaired titin function contributes to a sex-specific molecular mechanism. The functional derangements of titin are due to a sex-specific divergent regulation of PKG and CaMKII systems.

## Introduction

The prevalence of heart failure (HF), which is subdivided in heart failure with reduced (HFrEF), mildly reduced (HFmrEF) or preserved (HFpEF) ejection fraction, is increasing up to 3% by 2030 in the United States [1]. It is driven by the increase in the prevalence of HFpEF [2]. Although HF affects men and women equally, significant sex differences have been implicated. Apparently, the prevalence of HFpEF in women from 80 years of age is higher than in men [3]. In addition, the mortality of HF increases at all ages with comorbidities [3]. Accordingly, risk factors also differ according to HF subtype. Thus, patients with HFpEF are older, more likely female with more comorbidities, such as hypertension, renal impairment, obesity or diabetes when compared to HFrEF, which predominantly affects males [4]. Hypertension, indeed, is the most common risk factor for the development of HF in women [5]. Female with HFpEF in addition show higher levels of total cholesterol than men [6–8]. In the epidemiological HF study from Olmsted County, the predominant cause of death in patients with HFpEF was non-cardiovascular, whereas in patients with HFrEF it was coronary heart disease. Although men and women experienced comparable age-adjusted all-cause mortality, cardiovascular mortality was higher in male compared to female patients, whereas HF hospitalization rates were lower in women than in men [9]. Importantly, HF hospitalizations tend to increase, particularly in women, which may be due to the later onset and limited efficacy of therapies in HFpEF [2]. Neurohormonal antagonists have been shown to improve survival in HFrEF patients but were not efficient in HFpEF patients. Finding efficient treatments are very challenging due to the pathophysiological and clinical heterogeneity and to sex-specificities of HF [10].

Accordingly, an emerging number of studies support now sex differences in HF in terms of pathomechanism, adverse and reverse remodeling, as well as the clinical course and management, even with optimal medical therapy as recommended in recent guidelines [5]. Thus, HFpEF patients seem to exhibit a higher vascular and ventricular, endothelial dysfunction with impaired NO signaling, oxidative stress and inflammation [7]. However, mechanistic and clinical evidence concerning sex differences is limited, because females are still under-represented in experimental studies and clinical trials.

Here we aimed to study the combined role of risk factors (age and sex) and comorbidity (hypertension) in the development and progression of HF [11]. To this end, we performed a 1-year follow-up study in female and male transgenic rats overexpressing renin with consequent hypertension. We demonstrate significant sex-dependent differences in vascular and cardiac adaptation to neurohumoral stimulation in the rat. One potential molecular mechanism is dysregulation of PKG (cGMP-dependent protein kinase) and CaMKII (Ca^2+^/calmodulin-dependent protein kinase II) signaling pathways, leading to altered phosphorylation of the contractile system and thereby cardiomyocyte dysfunction.

## Materials and methods

### Animal experiments

**Fig. 1:**
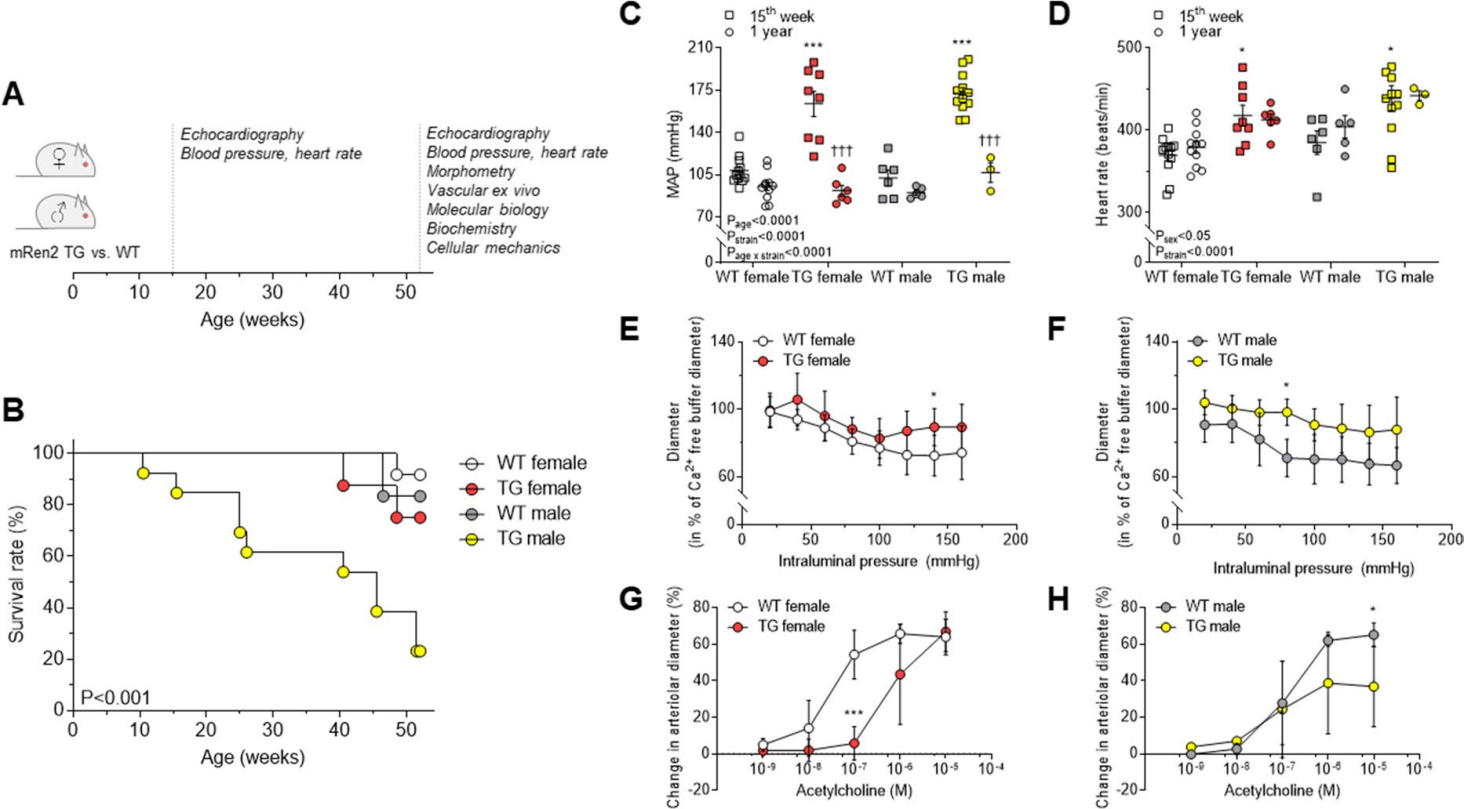
**A** Study design is shown. Dashed lines indicate ages at 15^th^ week and 1 year when experiments were done. TG: transgenic; WT: wild type. **B** One year mortality is shown as survival rate. **C** Mean arterial pressure (MAP) is shown at 15^th^ week and 1 year of age. **D** Heart rate is shown in 15-week- and 1-year-old rats. **E** Myogenic tone of skeletal muscle arteries is indicated in 1-year-old female rats. **F** Myogenic tone of skeletal muscle arteries is shown in males at 1 year of age. **G** Endothelial function of mesenteric arteries is indicated in 1-year-old female rats. **H** Endothelial function of mesenteric arteries is shown in males at 1 year of age. Data are given as mean ± SEM (panels C-D) or SD (panels E-H). On panels C-D: 15^th^ week of age: WT female: *n* = 12; TG female: *n* = 8; WT male: *n* = 6; TG male: *n* = 12; 1 year of age: WT female: *n* = 11; TG female: *n* = 6; WT male: *n* = 5; TG male: *n* = 3. On panels E-H: *n* = 3-4/group for males; *n* = 5-7/group for females. * *P*<0.05, *** *P*<0.001 vs. same-sex, same-age WT; ††† *P*<0.001 vs. same-group 15^th^ week.

All animal care and experimental procedures were approved by the Ethical Committee of the University of Debrecen (Ethical Statement number: 1/2013/DE MÁB) and were conform to the Directive 2010/63/EU of the European Parliament. Female and male homozygous rats carrying the mouse Ren-2 renin gene (mRen2) were obtained from the Max Delbrück Centrum Für Molekulare Medizin (Max Delbrück Center for Molecular Medicine in the Helmholtz Association (MDC), Berlin-Buch, Germany). Homozygous transgenic mRen2 rats were cross-bred with Sprague-Dawley (SD) rats (Wobe Ltd., Budapest, Hungary) as this was the original control rat strain in which the mouse Ren-2 gene was introduced [12]. Further inbred colonies - with unbiased birth rates - of 2^nd^ and 3^rd^ generations from heterozygous transgenic animals served as our study population for follow-up. The non-invasive phenotypic selection was used to include non-transgenic wild type (WT; female: *n* = 12; male: 6) and homozygous transgenic (TG; female: *n* = 8; male: *n* = 13) rats. Blood pressure (BP) and heart rate were monitored by a non-invasive CODA tail-cuff method (Kent Scientific Corp., Torrington, CT, USA). Body temperature was 37 ± 0.5 °C upon measurements. Mean arterial pressure (MAP) was calculated as follows: MAP = diastolic BP + (systolic BP – diastolic BP) / 3. No medication (e.g. anti-hypertensive drug) was administered to study subjects, and animals were fed a standard chow and tap water *ad libitum*. For study design see Fig. 1A. Experiments were carried out in rats at 15 weeks (WT female: *n* = 12; TG female: *n* = 8; WT male: *n* = 6; TG male: *n* = 12) and 1 year of age (WT female: *n* = 11; TG female: *n* = 6; WT male: *n* = 5; TG male: *n* = 3). Spontaneously surviving animals until 1 year of age were sacrificed with an intraperitoneal injection of thiopental (100 mg/body weight kg; B. Braun, Melsungen, Germany). After total anesthesia was achieved, animals were exsanguinated via a transverse cut on the thoracic aorta. Hearts, kidneys, livers and lungs were quickly excised and weighed, then, hearts were further dissected in relaxing solution (MgCl_2_: 1.0 mM; KCl: 100 mM; EGTA: 2.0 mM; ATP: 4.0 mM; imidazole: 10 mM; pH 7.0; all chemicals from Sigma-Aldrich, St. Louis, MO, USA), snap frozen in liquid nitrogen and stored at -80 °C until further use.

### Echocardiography

Transthoracic echocardiography with a General Electric Vivid E9 ultrasound system equipped with a linear 14.1 MHz i13L probe (General Electric, Fairfield, CT, USA) was performed on 15-week- and 1-year-old rats [13] under light anesthesia by a combination of ketamine and xylazine (50 mg/kg and 5 mg/kg body weight, respectively). Parasternal long axis M-mode was obtained at the level of the papillary muscles to assess left ventricular (LV) morphology (wall thickness and internal diameter) and systolic function such as ejection fraction (EF). Colour Doppler mode in apical four-chamber view was applied to estimate LV diastolic parameters at the level of the mitral valve. Early diastolic filling peak velocity (E), late filling peak velocity (A), E-wave deceleration time (DT) and isovolumetric relaxation time (IVRT) were recorded by pulsed-wave Doppler. LV myocardial performance index (MPI, i.e. Tei index) was subsequently calculated as follows: MPI = (IVCT + IVRT) / ET [14]. Images were analysed offline by EchoPAC clinical workstation software (General Electric).

### Vascular experiments

Myogenic tone and endothelial function were tested as described elsewhere [15]. Upon extermination, skeletal muscle and mesenteric arcade were collected and quickly transferred to ice-cold, oxygenated, Ca^2+^-free Krebs–Henseleit buffer (NaCL: 110 mM; KCL: 5.0 mM; MgSO_4_: 1.0 mM; KH_2_PO_4_ 1.0; glucose: 5.0; NaHCO_3_ 24 mM; pH 7.4; all from Sigma-Aldrich). The proximal caudal femoral artery (passive diameter: 200–250 μm) was dissected from the skeletal muscle (*m. gracilis*). From the mesenteric arcades, the 2^nd^-order-segment mesenteric artery was collected (passive diameter: 300–400 μm).

The skeletal muscle and mesenteric vessels were transferred to the organ bath of a pressure myograph (CH-1, Living Systems Instrumentation, St. Albans City, VT, USA). After cannulating the vessels, the buffer was replaced with Ca^2+^ containing (2.5 mM) Krebs–Henseleit buffer. The cannulated vessels were then allowed to equilibrate for 60 min at 37 °C under 80 mmHg pressure with a constant gas flow (85% N_2_, 10% O_2_, 5% CO_2_) directed into the buffer. Smooth muscle function was tested with norepinephrine (1 nM to 10 μM; Sigma-Aldrich) on both mesenteric and skeletal muscle arteries. On the skeletal muscle vessels, the endothelial function was tested with acetylcholine (1 nM to 10 μM; Sigma-Aldrich) after the development of myogenic tone. Endothelium-mediated dilation was measured after the myogenic tone had developed in the skeletal muscle arterioles and following pre-constriction with norepinephrine (10 μM) in the mesenteric arteries. Vessels (one per animal) that had intact smooth muscle and endothelial responses were used for further experiments (*n* = 3-4/group for males; *n* = 5-7/group for females).

The endothelium was denuded by perfusing the vessel with air (30–60 s) followed by distilled water (60–120 s) in a subset of skeletal muscle and mesenteric arteries. The dilative effect of acetylcholine was measured before and after this process, and only those vessels that showed no dilation upon acetylcholine after the procedure were used for further experiments. All endothelium-denuded arteries exhibited at least 80% constriction when tested with 10 μM norepinephrine (compared to the response before denudation).

### Cardiomyocyte force measurements

For mechanical measurements, LV cardiomyocytes were isolated as described previously [13, 16]. In brief, deep-frozen (−80 °C) samples were mechanically disrupted and membrane-permeabilized by Triton X-100 detergent (0.5%; Sigma-Aldrich, St. Louis, MO, USA) for 5 min in relaxing solution at 4 °C. The cell suspension was then washed 5 times in relaxing solution.

Cardiomyocyte (*n* = total 26-30 random myocytes from 3-6 different hearts/group) Ca^2+^-independent passive force (F_passive_) was measured in a relaxing buffer at room temperature within a sarcomere length (SL) range between 1.8 and 2.4 μm as described before [16]. Single cardiomyocytes were selected under an inverted microscope (Zeiss Axiovert 135, 40x objective; Carl Zeiss AG, Oberkochen, Germany) and attached with silicone adhesive between a force transducer and a high-speed length controller (piezoelectric motor) as part of a "Permeabilized Myocyte Test System" (1600A; with force transducer 403A; Aurora Scientific, Aurora, Ontario, Canada). Force values were normalized to the myocyte cross-sectional area. Following baseline force measurements, cardiomyocytes were incubated for 40 min in a relaxing solution supplemented with either protein kinase G (PKG1α; 0.1 U/mL, batch 034K1336), cGMP (10 μM) and dithiothreitol (DTT; 6 mM) (all from Sigma-Aldrich) or Ca^2+^/calmodulin-dependent protein kinase II (CaMKIIδ; 0.6 μg/mL in the calmodulin-containing buffer; Merck Millipore, Burlington, MA, USA). Thereafter, F_passive_ measurements were again performed in relaxing solution at sarcomere length (SL) of 1.8–2.4 μm.

Ca^2+^-sensitivity of skinned cardiomyocytes (*n* = total 11 random myocytes from 3-6 different hearts/group) was measured as described previously [13, 16]. Cells were attached at each end to a stainless-steel insect needle connecting to either a high-speed length controller (Aurora Scientific) or a sensitive force transducer (SensoNor AS, Horten, Norway) at 15 °C. Subsequent cardiomyocyte isometric force generation was recorded and analysed at SL of 2.3 μm. Ca^2+^-dependent force production of a single cardiomyocyte was induced by transferring the preparation from relaxing to activating solutions (same compositions as relaxing solution but with different free Ca^2+^-concentrations obtained with Ca^2+^-EGTA buffers). Ca^2+^-concentrations were indicated as –log_10_[Ca^2+^] values; accordingly, the pCa of relaxing solution was 9, whereas the pCa range of activating solutions was 4.75–7.0. Plots indicating relative active forces of an individual cardiomyocyte at each pCa value were fitted by a specific sigmoidal function in Origin 6.0 analysis program (OriginLab, Northampton, MA, USA). pCa value for the half-maximal contraction indicated by pCa_50_ defines per se the Ca^2+^-sensitivity of the contractile machinery. Maximal Ca^2+^-activated force (F_max_) was induced at pCa 4.75 (*n* = total 12 random myocytes from 3-6 different hearts/group). Original force values were again normalized to the myocyte cross-sectional area. Finally, during Ca^2+^-contractions, a so-called release-restretch maneuver, i.e. slack test, was applied in order to estimate the rate constant of force redevelopment (*k*_tr_). Accordingly, maximal actin-myosin turnover constant *k*_tr_ was determined at pCa 4.75 (*k*_tr,max_).

### Western immunoblot

Small proteins from LV samples (*n* = total 6 different homogenates from 3 hearts/group) were separated using 12% and 15% sodium dodecyl sulfate (SDS)-polyacrylamide gel electrophoresis (PAGE). Western immunoblot was then performed [13, 16] using primary antibodies against CaMKIIδ (Thermo Fisher Scientific, Waltham, MA, USA; #PA5-22168; dilution 1:1,000), oxidized CaMKII at Met281/282 (GeneTex, Irvine, CA, USA; #GTX36254; dilution 1:1,000), phosphorylated CaMKII at Thr286 (Cell Signaling Technology, Danvers, MA, USA; #12716; dilution 1:1,000) - that is Thr287 in CaMKIIδ because of slightly different numbering between isoforms [17] -, PKG-1α (Cell Signaling; #13511; dilution 1:1,000) in the presence of either DTT or N-ethylmaleimide (NEM), cardiac troponin I (cTnI; Cell Signaling; #4002; dilution 1:1,000), phosphorylated cTnI at Ser23/24 (Cell Signaling; #4004; dilution 1:1,000), cardiac myosin binding protein C (cMyBP-C; Abcam, Cambridge, UK; #Ab133499; dilution 1:1,000), phosphorylated Ser/Thr (ECM Biosciences, Versailles, KY, USA; #PP2551; dilution 1:500), sarco/endoplasmic reticulum Ca^2+^-ATPase 2 (SERCA2; Santa Cruz Biotechnology, Dallas, TX, USA; #Sc-376235; dilution 1:1,000), phospholamban (PLB; Abcam; #Ab219626; dilution 1:1,000), ryanodine receptor (RYR; Santa Cruz; #Sc-376507; dilution 1:1,000), adenosine monophosphate (AMP)-activated protein kinase alpha (AMPKα; Cell Signaling; #5831S; dilution 1:1,000), phosphorylated AMPKα at Thr172 (Cell Signaling; #2535S; dilution 1:1,000) and GAPDH (Sigma-Aldrich, Taufkirchen, Germany; #G9545; 1:10,000). Primary antibody binding was visualized using a secondary horseradish peroxidase (HRP)-labeled (anti-rabbit antibody (Cell Signaling; #7074S, dilution 1:10,000) or anti-mouse antibody (Cell Signaling; #7076S, dilution 1:10,000) IgG-HRP; dilution 1:10,000) and enhanced chemiluminescence (Clarity Western ECL Substrate; Bio-Rad, Hercules, CA, USA).

Titin was separated from LV samples (*n* = total 6 different homogenates from 3 hearts/group) by agarose-strengthened 1.8% SDS-PAGE, thereafter Western immunoblot was performed to measure site-specific phosphorylation of titin as previously described [16]. Phosphosite-specific anti-titin antibodies were custom-made by Eurogentec (Seraing, Belgium) with positions in N2Bus (N2B unique sequence) and PEVK (rich in proline, glutamate, valine and lysine amino acids) domains of the mouse (*Mus musculus*) titin according to UniProtKB identifier A2ASS6. The following rabbit polyclonal affinity purified antibodies were used: anti-phospho-N2Bus (Ser4043) against QELLS(PO3H2)KETLFP (dilution 1:100); anti-phospho-N2Bus (Ser4080) against LFS(PO3H2)EWLRNI (dilution 1:500); anti-phospho-PEVK (Ser12884) against KLRPGS(PO3H2)GGEKPP (dilution 1:500). The amino acid sequences of rat titin at Ser4043, Ser4080 and Ser12884 are identical to the amino acid sequences of mouse, and refer to human titin at Ser4062, Ser4099 and Ser12022, respectively [18]. Secondary HRP-labeled anti-rabbit antibody (Cell Signaling; #7074S, dilution 1:10,000) and enhanced chemiluminescence (Clarity Western ECL Substrate; Bio-Rad, Hercules, CA, USA) were used accordingly.

Western blot signals were visualized using the ChemiDoc Imaging System and analysed with Multi Gauge V3.2 software (both from FUJIFILM, Minato, Tokyo, Japan). Coomassie-based PVDF stains were saved for comparison of protein load for titin.

### Protein kinase activity assays

LV tissues samples (*n* = total 6 different homogenates from 3 hearts/group) were homogenized in 25 mM Tris-HCl (pH 7.4), 1 mM EDTA, 2 mM EGTA, 5 mM DTT, 0.05% Triton X-100 and protease inhibitor cocktail (all from Sigma-Aldrich) and centrifuged for 5 min. Supernatants containing equal amounts of total protein were analysed for PKG activity as described previously [16]. Briefly, reaction mixtures were incubated at 30 °C for 10 min. Reaction mixtures contained 40 mM Tris-HCl (pH 7.4), 20 mM Mg(CH_3_COO)_2_, 0.2 mM [^32^P] adenosine triphosphate (ATP) (500–1,000 cpm pM–1; Amersham, Little Chalfont, UK), 113 mg/mL heptapeptide (RKRSRAE), and 3 μM cGMP (both from Promega, Madison, WI, USA), and a highly specific inhibitor of cyclic adenosine monophosphate-dependent protein kinase (5–24; Calbiochem, San Diego, CA, USA). The reaction was terminated by spotting 70 μL onto Whatman P-81 filters (MACHEREY-NAGEL, Dueren, Germany). Samples were subsequently incubated and washed with 75 mM H_3_PO_4_ for 5 min to remove ATP and free phosphate. Filters were then washed with 100% ethanol and air-dried before quantification. PKG activity was quantified using a Wallac 1409 Liquid Scintillation Counter (Hidex Oy, Turku, Finland). Specific activity of PKG was expressed as pM of ^32^P incorporated into the substrate (pM/min/mg protein). Results of triplicate determinations were averaged.

CaMKII activity was determined using a CycLex CaMKII assay kit (CY-1173; MBL, Woburn, MA, USA) as described earlier [16]. LV tissue samples (*n* = total 6 different homogenates from 3 hearts/group) were homogenized in sample buffer containing 15% glycerol, 62.5 mM Tris (pH 6.8), 1% SDS, protease inhibitor and protein phosphatase inhibitor. Homogenates were centrifuged at 10,000 *g* for 15 min at 4 °C. The supernatant was removed and stored at −80 °C. Protein samples were loaded onto microliter wells coated with the CaMKII substrate, Syntide-2, along with kinase reaction buffer with or without Ca^2+^/calmodulin. To quantify CaMKII activity, a standard curve correlating the amount of active CaMKII and the level of phosphorylation of Syntide-2 was constructed. Accordingly, results of triplicate determinations were averaged and CaMKII activity was expressed as mU/mL.

### Angiotensin-converting enzyme (ACE) activity measurements

Experiments were performed as reported before [13]. Frozen cardiac tissue samples were homogenized (*n* = 3-9 different homogenates from 3 hearts/group) in 100 mM TRIS, pH 7.0 in a ratio of 1 mg wet tissue in 5 μl buffer on ice. PMSF (phenylmethylsulfonyl fluoride, 1 mM final concentration) and cOmplete™ protease inhibitor cocktail was added immediately after homogenization to inhibit protein degradation. Tissue homogenate was diluted to 1 mg/mg protein concentration by 100 mM TRIS-HCl, pH 7.0 (for ACE) or 75 mM TRIS-HCl, pH 6.5 (for ACE2) before the measurements. Tissue homogenates were then diluted 20-fold in the activity measurements.

ACE activity was measured by a kinetic fluorescent assay using the Abz-FRK(Dnp)-P substrate (excitation: 320; emission: 405 nm; dissolved in DMSO to 1 mM stock, final concentration was 15 μM). The activity measurement buffer also contained: 100 mM Tris-HCl, 50 mM NaCl, 10 μM ZnCl_2_.

ACE2 activity was measured by a kinetic fluorescent assay using the Mca-APK-(Dnp) substrate (excitation: 320; emission: 405 nm; dissolved in DMSO to 1 mM stock, final concentration was 50 μM). The activity measurement buffer also contained: 75 mM TRIS-HCl, pH 6.5, 500 mM NaCl, 10 μM ZnCl_2_,10 μM bestatin-hydrochloride, 10 μM Z-prolyl-prolinal (Enzo Life Sciences, Exeter, UK), 5 μM amastatin-hydrochloride, 10 μM captopril.

Assays were recorded for 60 min at 37 °C in the wells of a 96 well black plate in a BMG (BMG Labtech, Ortenberg, Germany) fluorescent plate reader (NovoStar or ClarioStar). The activity was expressed in units (U) representing 1 nM substrate cleavage in 1 min by 1 mg of protein. The specific activity represented about 95% of the total activity as tested by captopril (10 μM) for ACE or about 50% of the total activity as tested by MLN4760 (5 μM) for ACE2.

### Statistical analysis

Three-way ANOVA with Tukey post hoc test was used to compare same-group 1 year vs. 15^th^ week, same-sex TG vs. WT or same-strain male vs. female (in vivo data). The effects of in vitro protein kinase incubations (after vs. baseline) and sarcomere lengthening on F_passive_ were compared by paired t-test of repeated measurements accordingly. Otherwise data were compared by two-way ANOVA followed by Tukey's multiple comparisons test. Data are given as mean values ± SEM or SD. Statistical significance was assigned when *P*<0.05. Note, that only significant groups effects and interactions are indicated from multiple comparisons (i.e. three- and two-way ANOVA).

## Results

### TG animals showed sex-dependent mortality and vascular adaptation

We observed striking differences in survival rates (WT female: 91.7 %; TG female: 75 %; WT male: 83.3 %; TG male: 23.1 %) as TG male rats showed significantly higher mortality at 1 year than any other group (Fig. 1B). On the other hand, irrespective of sex, MAP (Fig. 1C) and heart rate (Fig. 1D) were higher in 15-week-old TG animals compared to WT, albeit blood pressure was nearly normal in 1-year-old TG animals.

The spontaneous myogenic tone of gracilis arteries reduced with increase in intraluminal pressure in all groups at 1 year of age, but appeared lower in TG vs. WT animals, irrespective of sex (Fig. 1E and F). Furthermore, endothelium function was monitored by measuring the acetylcholine responses. Acetylcholine triggers endothelium-dependent vasodilation. In TG rats, endothelial dysfunction was observed which differed dependent on sex (Fig. 1G and H). Thus, in TG female animals, acetylcholine sensitivity was decreased (Fig. 1G) as the EC_50_ of acetylcholine (778 nM) was significantly higher than that of WT female rats (49 nM). However, in male rats the EC_50_ was similar in TG (637 nM) and WT (630 nM; Fig. 1H), whereas in TG male animals the maximal vasodilation upon acetylcholine treatment was significantly reduced (36.9 ± 12.6 %) as compared to WT group (65.2 ± 3.7 %; Fig. 1H). In contrast, in TG females the maximal vasodilation capacity in response to acetylcholine was preserved (66.9 ± 4.9 %) compared to the female WT group (64.1 ± 4.0 %; Fig. 1G).

### The TG rats showed sex-specific cardiac dysfunction and remodeling

**Fig. 2:**
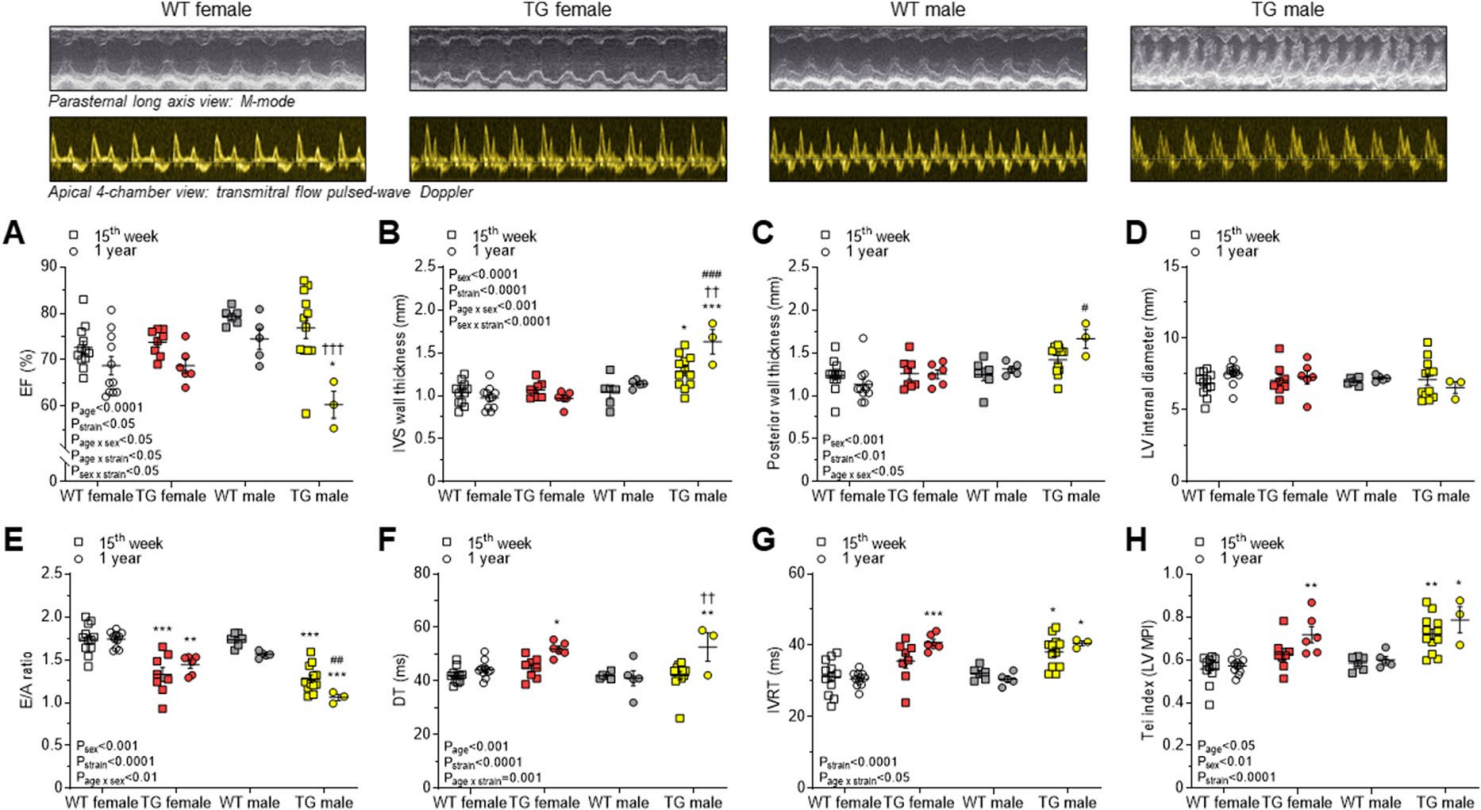
Echocardiography data are shown at 15^th^ week and 1 year of age as follows: **A** Ejection fraction (EF); **B** Interventricular septum (IVS) wall thickness; **C** Posterior wall thickness; **D** Left ventricular (LV) internal diameter in diastole; **E** Ratio of early (E) and late (A) diastolic filling peak velocity by pulsed-wave Doppler; **F** E-wave deceleration time (DT); **G** Isovolumetric relaxation time (IVRT); **H** LV myocardial performance index (MPI), i.e. Tei index. Insets are corresponding representative echocardiographic images at 1 year of age. Data are shown as mean ± SEM. 15^th^ week of age: WT female: *n* = 12; TG female: *n* = 8; WT male: *n* = 6; TG male: *n* = 12; 1 year of age: WT female: *n* = 11; TG female: *n* = 6; WT male: *n* = 5; TG male: *n* = 3. * *P*<0.05, ** *P*<0.01, *** *P*<0.001 vs. same-sex, same-age WT; # *P*<0.05, ## *P*<0.01, ### *P*<0.001 vs. same-strain, same-age female; †† *P*<0.01, ††† *P*<0.001 vs. same-group 15^th^ week.

The ejection fraction (EF) of TG rats was preserved in both sexes at 15 weeks of age (Fig. 2A). However, in 1-year-old TG rats fractional shortening (data not shown) and EF were reduced significantly exclusively in males (Fig. 2A). Likewise, LV hypertrophy as determined by interventricular septum and posterior wall thicknesses was found only in TG male animals already at the age of 15 weeks becoming more severe over 1 year (Fig. 2B and C). LV internal diameter remained nearly constant at the age of 15 weeks and 1 year for both sexes, indicating that no dilation of the LV occurred (Fig. 2D).

In addition, we observed a decreased ratio of early and late filling velocities (E/A ratio) reflecting an impaired relaxation in 15-week- and 1-year-old TG female and male rats compared to WT animals (Fig. 2E). In 1-year-old TG animals we saw a progression in relaxation abnormalities as shown by significantly prolonged deceleration time (DT) of the early filling velocity (Fig. 2F) and isovolumetric relaxation time (IVRT; Fig. 2G). Interestingly, impairment of relaxation was advanced earlier in TG males than in females as IVRT in TG males was already prolonged at 15 weeks of age (Fig. 2G). Accordingly, the Tei index reflecting myocardial performance was impaired in TG males earlier than in TG females, namely in 15-week-old rats, while it was equally impaired in males and females in the TG vs. WT groups at 1 year (Fig. 2H).

In line with echocardiography, cardiac enlargement and LV hypertrophy were present only in the TG male group at 1 year evidenced by significantly higher heart weight/TL and LV weight/TL ratios than those in either WT males or TG females (Table 1.). Pulmonary congestion could not be confirmed in TG animals because lung wet/dry weight ratios did not reach the level of statistical significance (Table 1.). Liver wet/dry weight ratios were found to be similar as well (Table 1.).

**Table 1:** Morphometry in 1-year-old animals is shown

	WT female	TG female	WT male	TG male
Heart weight (mg) / TL (mm)	27.21±1.40	29.90±2.63	34.55±1.96 ^#^	46.84±2.29 * ^###^
LV weight (mg) / TL (mm)	19.84±0.93	20.37±0.97	23.23±0.50	30.02±1.13 ** ^###^
Lung wet / dry weight	4.82±0.18	5.17±0.32	4.67±0.20	5.43±0.29
Liver wet / dry weight	2.93±0.11	3.08±0.06	3.00±0.14	2.95±0.14

*TL* tibial length. Data are given as mean ± SEM. WT female: *n* = 11; TG female: n = 6; WT male: n = 5; TG male: n = 3. * *P*<0.05, ** *P*<0.01 vs. same-sex WT; # *P*<0.05, ### *P*<0.001 vs. same-strain female. Heart weight: *P*sex<0.0001; *P*strain<0.01; *P*sex x strain<0.05. LV weight/TL: *P*sex<0.0001; *P*strain<0.01; *P*sex x strain<0.05

### Myofilament Ca^2+^-dependent active force production and protein phosphorylation showed sex-dependent differences in both WT and TG rats

**Fig. 3:**
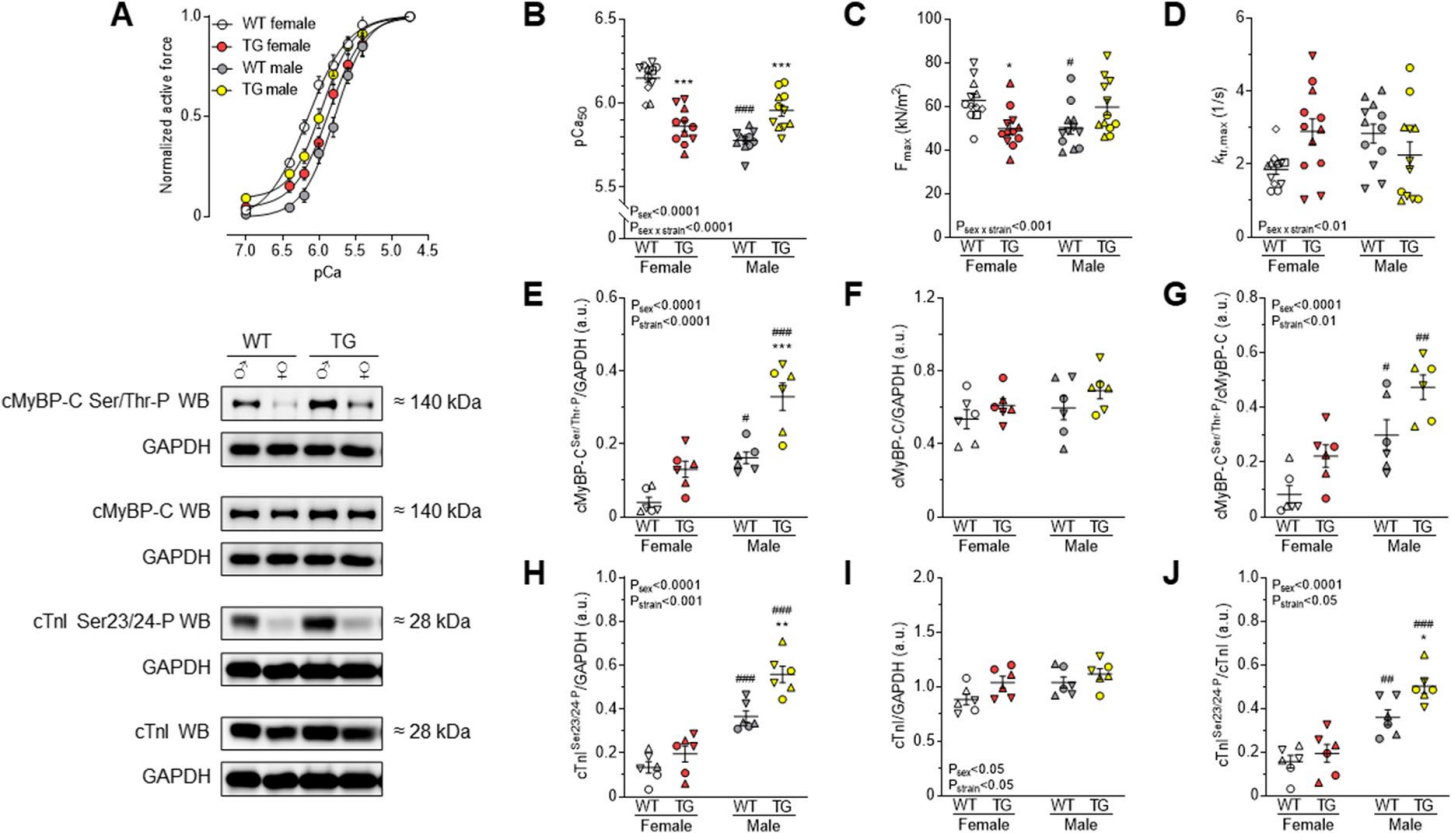
Cardiomyocyte active force production is demonstrated as follows: **A** Normalized active force vs. pCa relationships; **B** pCa_50_ values describing Ca^2+^-sensitivity of force production; **C** Maximal Ca^2+^-activated force at pCa 4.75 (F_max_); **D** Maximal rate constant of force redevelopment (*k*_tr,max_) at pCa 4.75. **E** Phosphorylation (P) level of cMyBP-C at Ser/Thr residues is normalized to GAPDH. **F** Relative to GAPDH, cMyBP-C expression is shown. **G** P of cMyBP-C is normalized to cMyBP-C protein amount. **H** Site-specific P of cTnI at Ser23/24 is normalized to GAPDH. **I** Relative to GAPDH, cTnI expression is shown. **J** Ser23/24-P of cTnI is normalized to cTnI protein amount. Insets are corresponding representative western blot (WB) images. Data are given as mean ± SEM. On panels A-B: *n* = total 11 random myocytes from 3-6 different hearts/group. On panels C-D: *n* = total 12 random myocytes from 3-6 different hearts/group. On panels E-J: *n* = total 6 different homogenates from 3 hearts/group. On panels B-J similar plot symbols indicate samples from identical hearts. * *P*<0.05, ** *P*<0.01, *** *P*<0.001 vs. same-sex WT; # *P*<0.05, ## *P*<0.01, ### *P*<0.001 vs. same-strain female

**Fig. 4:**
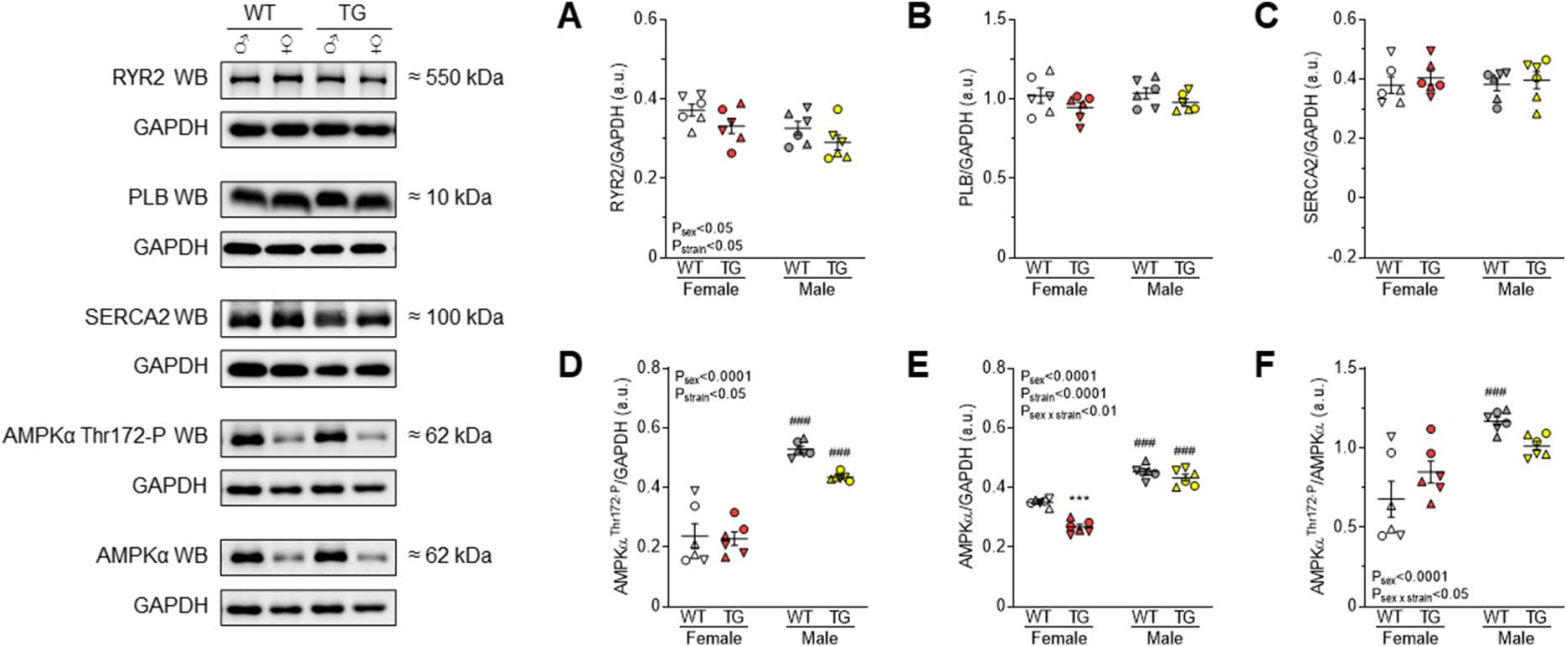
Ca^2+^ handling protein expression is shown normalized to GAPDH as follows: **A** RYR2; **B** PLB; **C** SERCA2. **D** P level of AMPK at Ser/Thr residues. **E** AMPK. **F** P of AMPK is normalized to AMPK protein amount. Insets are corresponding representative WB images. Data are shown as mean ± SEM. *n* = total 6 different homogenates from 3 hearts/group. Similar plot symbols indicate biological replicates. *** *P*<0.001 vs. same-sex WT; ### *P*<0.001 vs. same-strain female

In TG rats, neurohormonal activation responses and progression towards HF were more prominent in males compared to females. Specifically, the investigation of cardiac function at the cellular level revealed sex-dependent differences in myofilament Ca^2+^-dependent active force production and protein phosphorylation in both WT and TG rats. The normalized active force vs. pCa relationships clearly demonstrated sex-dependent differences within TG animals. Males had increased pCa_50_ values, while females had decreased values compared to the appropriate WT groups (Fig. 3A-B). The maximal force (F_max_) showed lower levels in WT males and slightly higher levels in TG males, although not significantly. TG females, on the other hand, exhibited decreased F_max_ compared to the appropriate WT groups (Fig. 3C). Differences in the maximal rate constant of force redevelopment, *k*_tr,max_, did not reach the level of significance (Fig. 3D). Phosphorylation levels of cMyBP-C and cTnI, which are crucial for regulating contractility, were higher in WT males compared to WT females and further increased in TG males (Fig. 3E-J). However, cMyBP-C and cTnI phosphorylation did not significantly change in TG females (Fig. 3E-J). The expression levels of Ca^2+^ handling proteins in cardiomyocytes, such as RYR2 (Fig. 4A), PLB (Fig. 4B) which regulates the activity of SERCA, and SERCA2 (Fig. 4C), were similar in female and male WT and TG animals.

### Neurohormonal activation responses and progression towards HF were more prominent in TG male vs. female rats

Furthermore, there was a selective increase in LV ACE activity in male TG rats, but tissue ACE activities in kidneys and lungs remained unchanged (Table 2). Regardless of sex, tissue ACE2 activities in LV, kidneys, and lungs did not significantly differ between TG and WT groups (Table 2). AMPK, which acts as an energy and redox sensor, showed higher expression levels and activating phosphorylation in males compared to females (Fig. 4D-F). Neurohumoral over-activation in TG animals resulted in a non-significant (*P*=0.058) decrease in AMPK phosphorylation exclusively in males, contributing to disease development (Fig. 4D). In females, the expression level of AMPK was reduced, and the ratio of phosphorylated AMPK to total AMPK showed a slight increasing trend (Fig. 4E). These findings highlight the sex-specific aspects of the pathomechanism, emphasizing the complex role of sex in cardiac responses to neurohormonal activation and myofilament protein alterations.

**Table 2:** Tissue angiotensin-converting enzyme (ACE) and ACE2 activities are indicated at 1 year of age

	WT female	TG female	WT male	TG male
ACE activity (U/mg)				
*LV*	4.92±0.36	5.39±0.63	5.31±0.25	9.28±0.82 ** ^##^
*Lung*	61.1±6.1	59.8±5.3	69.4±12.1	75.3±5.5
*Kidney*	15.91±2.28	16.70±2.41	6.48±3.49	15.81±2.47
ACE2 activity (U/mg)				
*LV*	1,947±135	1,627±185	2,377±492	2,481±606
*Lung*	20,774±1,666	21,674±2,252	16,511±3,991	12,692±3,508
*Kidney*	8,615±875	8,525±1,890	6,027±2,478	10,154±1,555

Data are given as mean ± SEM. *n* = 3-9 different homogenates from 3 hearts/group. ** *P*<0.01 vs. same-sex WT; ## *P*<0.01 vs. same-strain female. LV ACE activity: *P*sex<0.01; *P*strain<0.01; *P*sex x strain<0.05

### Oxidative modifications of PKG and CaMKII lead to increased cardiomyocyte passive stiffness in TG rats

**Fig. 5:**
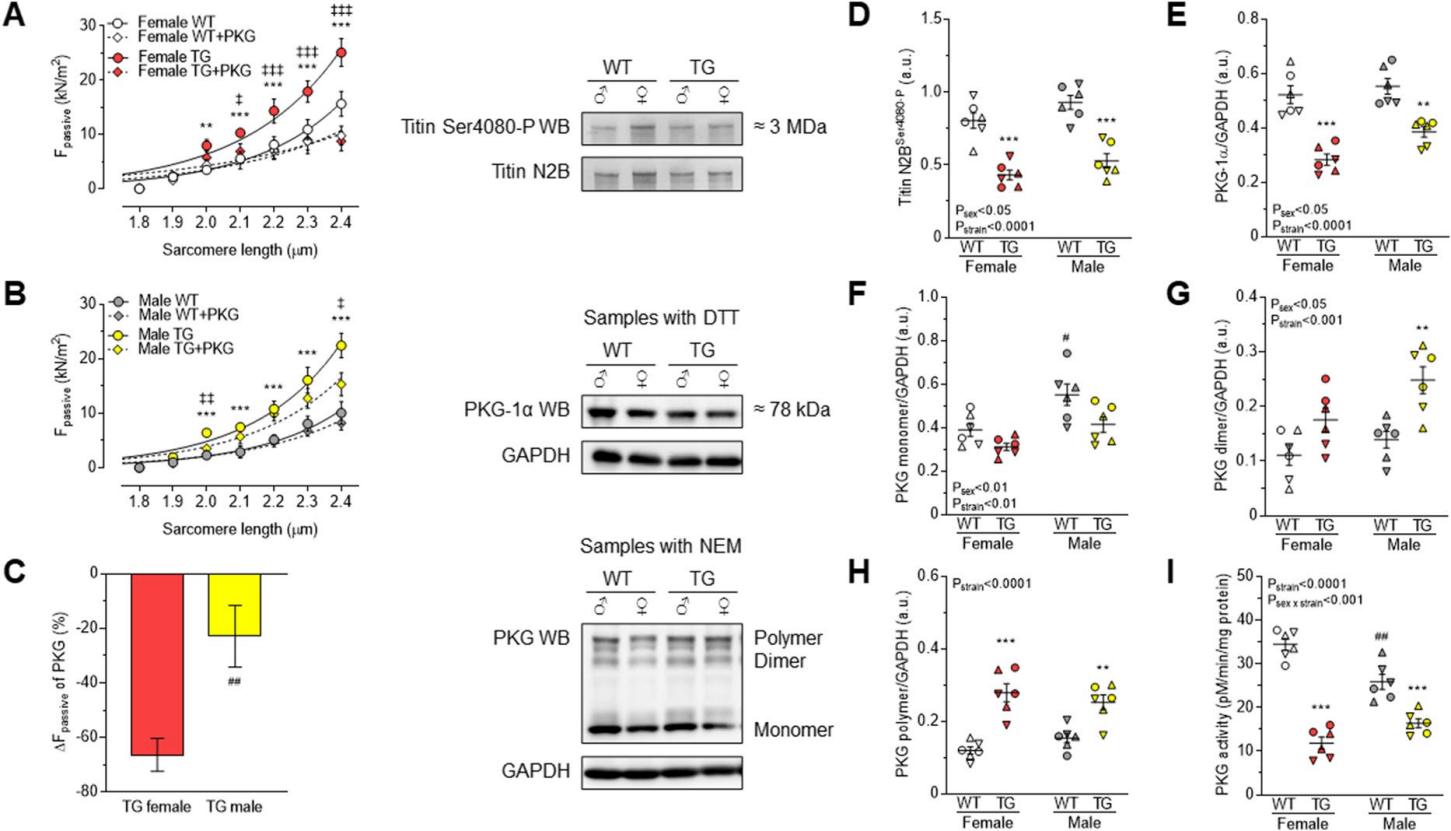
**A** Cardiomyocyte Ca^2+^-independent passive force (F_passive_) vs. sarcomere length relationships are shown in females WT and TG before and after incubation with PKG. **B** Cardiomyocyte passive stiffness is indicated in males WT and TG before and after in vitro PKG treatment. **C** Comparison of passive stiffness lowering effect (∆F_passive_) of PKG in TG females vs. TG males is shown at sarcomere length of 2.4 μm. **D** Site-specific P at Ser4080 of titin N2B isoform. **E** PKG-1α level is normalized to GAPDH in the presence of dithiothreitol (DTT). **F** PKG monomers; **G** PKG dimers; and **H** PKG polymers are normalized to GAPDH in samples supplemented with N-ethylmaleimide (NEM). **I** PKG activity is shown. Insets are corresponding representative WB images. Data are given as mean ± SEM. On panels A-C: *n* = total 13-15 random myocytes from 3-6 different hearts/group. On panels D-I: *n* = total 6 different homogenates from 3 hearts/group. On panels D-I similar plot symbols indicate samples from identical hearts. ** *P*<0.01, *** *P*<0.001 vs. same-sex WT; # *P*<0.05, ## *P*<0.01 vs. same-strain female; ‡ *P*<0.05, ‡‡ *P*<0.01, ‡‡‡ *P*<0.001 TG+PKG vs. TG baseline.

The passive stiffness of cardiomyocytes is regulated by the sarcomeric giant protein titin. The passive stiffness of cardiomyocytes is deranged in heart disease. Thus, irrespective of sex, we observed increased cardiomyocyte passive stiffness in the TG vs. WT groups over the range of sarcomere length of 2.0-2.4 μm in both females (Fig. 5A) and males (Fig. 5B). Treatment of isolated skinned TG cardiomyocytes with PKG restored the passive stiffness in female TG cardiomyocytes. In male TG cells PKG treatment also reduced the passive stiffness, though incomplete (Fig. 5A-C). Since passive stiffness is partly dependent on the phosphorylation state of titin, in which PKG and CaMKII are involved, we at first determined PKG-dependent titin phosphorylation at Ser4080. The phosphorylation of this site was reduced in both sexes of TG vs. WT groups (Fig. 5D) probably due to decreased PKG expression (Fig. 5E and F) and activity (Fig. 5I). Moreover, PKG expression level (Fig. 5E) was even lower in TG females vs. males (*P*=0.054). A loss in activity might be caused by oxidation of PKG which induces dimerization or polymerization of PKG, which can be reversed by the addition of DTT. Levels of PKG monomers and dimers or polymers have been analysed in TG and WT animal cardiac tissue. In general, the level of PKG monomers were clearly higher in males vs. females in WT rat cardiomyocytes (Fig. 5F). Nonetheless, levels of PKG monomers were non-significantly lower in TG rats compared to WT rats, whereas levels of PKG dimers and polymers significantly increased in TG animals (Fig. 5F-H), indicating oxidative modification of PKG in TG animals. These observations match the lower PKG activity in TG females and males.

**Fig. 6:**
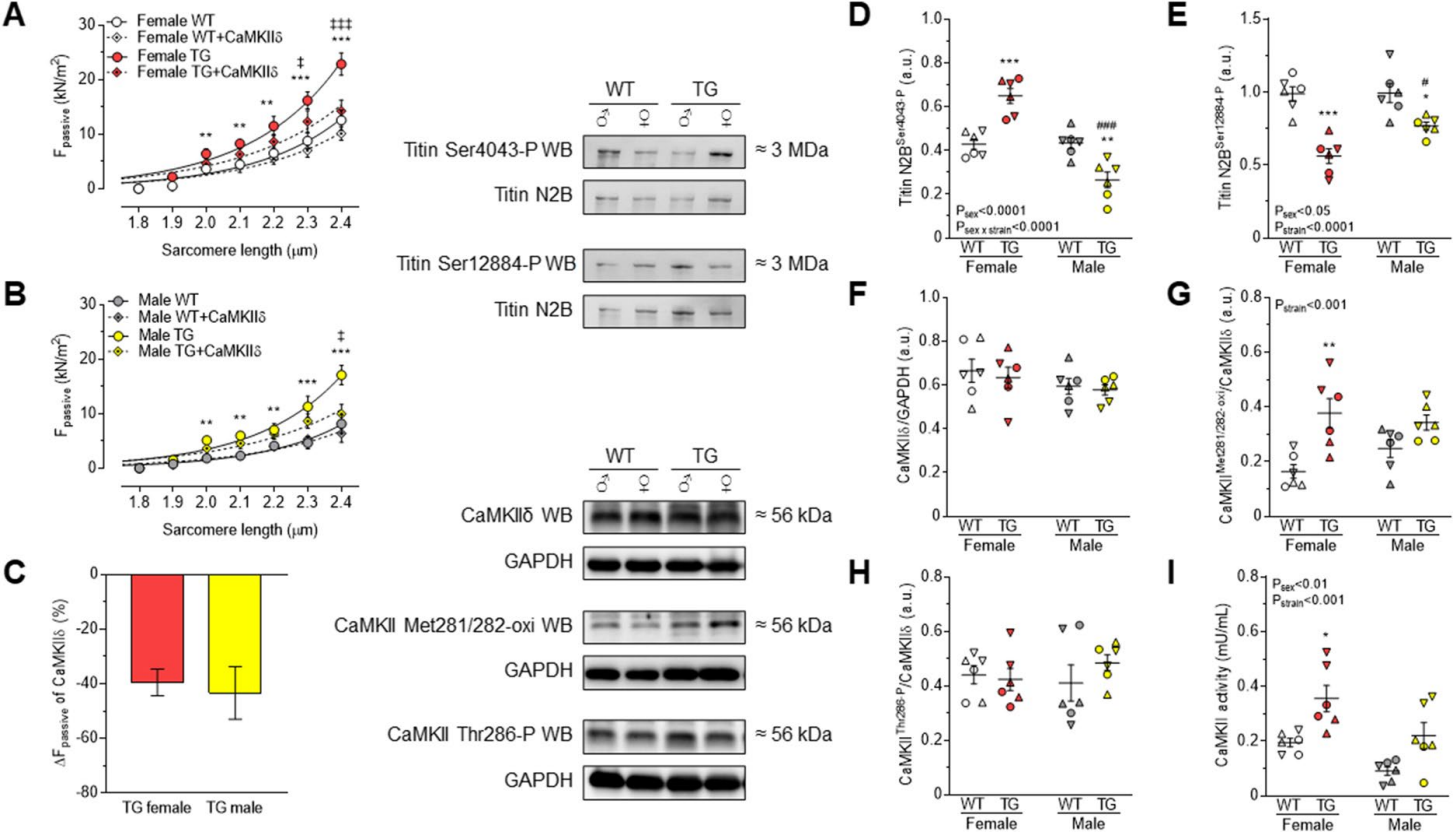
**A** Cardiomyocyte passive stiffness is indicated in females WT and TG before and after in vitro CaMKIIδ treatment. **B** Cardiomyocyte passive stiffness is shown in males WT and TG before and after incubation with CaMKIIδ. **C** Comparison of passive stiffness lowering effect (∆F_passive_) of CaMKIIδ in TG animals is shown at sarcomere length of 2.4 μm. Site-specific P of titin N2B isoform is indicated as follows: **D** at Ser4043; **E** at Ser12884. **F** Relative to GAPDH, total CaMKIIδ expression is shown. **G** Oxidation level of CaMKII at Met218/282 is normalized to total CaMKIIδ. **H** Site-specific P at Thr286 of CaMKII is normalized to total CaMKIIδ protein amount. **I** CaMKII activity is indicated. Insets are corresponding representative WB images. Data are shown as mean ± SEM. On panels A-C: *n* = total 13-15 random myocytes from 3-6 different hearts/group. On panels D-I: *n* = total 6 different homogenates from 3 hearts/group. On panels D-I similar plot symbols indicate samples from identical hearts. * *P*<0.05, ** *P*<0.01, *** *P*<0.001 vs. same-sex WT; # *P*<0.05, ### *P*<0.001 vs. same-strain female; ‡ *P*<0.05, ‡‡‡ *P*<0.001 TG+CaMKIIδ vs. TG baseline

As the effect of PKG to the isolated skinned TG cardiomyocytes, CaMKIIδ also reduced the passive stiffness to a similar extent in females and males (Fig. 6A-C), but it was less effective than PKG treatment. CaMKIIδ-dependent titin phosphorylation differs dependent on the phosphorylation site and sex. Thus, titin phosphorylation at Ser4043 was higher in TG females, while it was lower in males (Fig. 6D). However, hypophosphorylation at Ser12884 of titin was observed in both TG females and males with a relatively higher level in males than in females (Fig. 6E). Despite these differences in phosphorylation, CaMKIIδ expression (Fig. 6F) and its phosphorylation at Thr286 remained unchanged (Fig. 6H). However, CaMKII oxidation at Met281/282 was significantly higher only in TG females as compared to WT females (Fig. 6G). Accordingly, CaMKII activity was exclusively increased in TG females (Fig. 6I). In contrast to TG females, oxidation (Fig. 6G) and activity of CaMKII (Fig. 6I) did not significantly change in TG males. Of note, CaMKII activity was lower in WT males than that in WT females, but this difference did not reach the level of statistical significance in TG rats (Fig. 6I).

In summary, we observed differences between females and males on all levels, concerning heart and vascular function, neurohumoral responses, contractile function of cardiomyocytes, phosphorylation status of regulatory sarcomeric proteins and regulation of passive stiffness.

## Discussion

Here we demonstrate sex-dependent alterations in cardiovascular adaptation, consequently, significant differences in the survival of aged hypertensive rats during their progression to HF were observed. Male TG animals predominantly showed characteristics of the HFrEF phenotype associated with higher mortality, hypertrophy and neurohormonal activation compared to female TG animals. In contrast, female TG rats showed characteristics of the HFpEF phenotype. Sex-specific heterogeneity could be observed in vascular endothelial and cardiomyocyte function. Our data also highlighted a potential mechanism for sex-dependent differences in cardiac protein kinase-mediated phosphorylation, which affects the active and passive function of the cardiac sarcomere.

In line with our experimental data, the Swedish Heart Failure Registry study reported a higher risk of HFrEF in males, while HFpEF was more common in old females with comorbidities. But the adjusted prognosis was better in females regardless of EF [19]. Similar sex differences were found in 9-month-old mRen2 vs. SD control rats [20]. Risk factors of HFrEF, such as blood pressure, cardiac enlargement and plasma lipid levels (e.g. LDL-C) were higher in male vs. female TG animals. Likewise, in hypertensive Dahl salt-sensitive rats with signs of HFpEF, males compared to females exhibited more LV dilation with worse survival and a higher risk of sudden cardiac death [21]. Our current work showed diastolic dysfunction by echocardiography in mRen2 females, while the LV ejection fraction and heart morphology were preserved. In contrast, TG males showed a reduction of EF at 1 year on top of LV hypertrophy.

Moreover, local activation of the renin-angiotensin-aldosterone system (RAAS) showed the same age- and sex-dependent pattern. The trend of increased tissue ACE activity in the LV of homozygous TG vs. SD males at 15 weeks [13] became significantly higher at 1 year but remained unchanged in age-matched females. Interestingly, LV ACE2 activity, which was decreased in 15-week-old TG males [13], was unaltered in 1-year-old TG male and female rats. In former reports suppression of baroreflex gain and associated end-organ damage were more pronounced in mRen2 males compared with mRen2 females. The conclusion was drawn that females had relative protection from autonomic dysfunction and end-organ damage in the mRen2 model [22]. In this regard, the protective role of oestrogen has been repeatedly demonstrated in diastolic dysfunction of the premenopausal heart [23]. This concept was also proven in ovariectomized mRen2 female (Lewis) rats, showing the activation of the tissue RAAS and nitric oxide synthase (NOS) system being responsible for the production of reactive oxygen species (ROS) at low oestrogen levels [23]. This observation is in line with our recent findings showing that female TG rats exhibit more oxidative stress than male TG rats at the age of 1 year [24].

AMPK as a cardiac energy and redox sensor has beneficial effects on the heart. AMPK can be activated by unconventional pathways, including ischemia [25], hypoxia [26], oxidant signaling [27], hormones [28], DNA damage [29] and cytokines [30]. Its activation upon pressure overload, ischemia, metabolic and oxidative stress protects the heart from ROS and failure [31]. Of note, endothelial AMPKα2 isoform attenuates ACE and thereby prevents angiotensin II formation [32]. In this study, males presented higher phosphorylation and expression levels, i.e. higher activity of cardiac AMPK, independently of neurohumoral activation. In TG males an apparent hypophosphorylation of AMPK was observed, indicating lower AMPK activity and loss of cardiac protection. Female TG rats showed a lower expression level of AMPK and a slightly higher AMPK activation as revealed from the ratio of AMPK phosphorylation over AMPK total. Increased AMPK activation in TG female could be due to the fact that TG female have a higher oxidative stress than TG male as we recently have shown [24]. Mechanistically, it has been shown that AMPK activation reduces oxidative stress through acting on nicotinamide adenine dinucleotide phosphate (NAD(P)H) oxidases (Nox) mitochondria and antioxidant enzyme expression and thereby playing a significant role in controlling oxidative stress [31]. The same mechanism might occur in TG female rats to control the massive increase of oxidative stress [24].

Microvascular alterations are not unique to HFpEF but are also present in comorbidities associated with HFpEF, including hypertension. Our results demonstrate impaired myogenic tone in the gracilis arteries, which was further decreased in hypertensive animals. These findings are consistent with the research conducted by Toth et al., who demonstrated the detrimental effects of age and hypertension on cerebral autoregulation [33]. These findings further emphasize the systemic impact of aging on vascular health, as previously suggested by Csipo et al. [34]. Although coronary microvascular dysfunction is highly prevalent in both men and women with HFpEF, the drivers of microvascular dysfunction might differ by sex [35]. Acetylcholine-induced vasorelaxation is an endothelium-dependent mechanism and is closely dependent on oxidative stress [36]. Here we observed striking differences in endothelial dysfunction of male and female TG rats. In line with our results, impaired acetylcholine-induced relaxation of aortic segments and reduced basal NO were reported in a HFpEF model of female senescence-accelerated prone mice [37]. Of note, blood pressure was normal in TG rats at 1 year of age and was associated with lower spontaneous arterial myogenic tone in both sexes of the TG group. It is worth noting that low blood pressure is a common occurrence in advanced HF and can pose challenges in terms of optimal patient management [38].

Furthermore, TG rats showed sex specific changes in isolated skinned cardiomyocytes. Cardiomyocytes from TG female animals exhibited decreased Ca^2+^-sensitivity of force production and decreased maximum tension (F_max_), while Ca^2+^-sensitivity increased in TG males when compared to their corresponding littermates. These distinct changes were associated with altered phosphorylation of myofilament proteins that are responsible for Ca^2+^-dependent force production [39, 40]. We found hyperphosphorylated cMyBP-C and cTnI exclusively in TG males. It has been reported that phosphorylation of Ser282 in cMyBP-C presumably by CaMKII might be prerequisite for subsequent phosphorylation of other sites [41]. However, CaMKII activity was increased only in our TG female animals which maybe could promote phosphorylation of other sites to modulate Ca^2+^-sensitivity at low Ca^2+^ levels [42]. Phosphorylated cTnI at Ser23/Ser24 has been shown to reduce myofilament Ca^2+^-sensitivity [43]. Ser23/Ser24 sites of cTnI are substrates for multiple protein kinases, including protein kinase A (PKA) and D1 (PKD), PKG and protein kinase C (PKC) isoforms [40]. However, PKC-dependent phosphorylation at cTnI Ser43/45 results in enhanced myofilament Ca^2+^-sensitivity [43]. Hence, each phosphorylation site might have distinct impact, and the regulation of cardiac contractility requires a balanced synergy between the different opposing effects. It is important to note that CaMKII-dependent cTnI phosphorylation is involved in frequency-dependent myofilament Ca^2+^ desensitization [44], likewise, PKGIα phosphorylates cMyBP-C several sites which may occur in pressure overload, i.e. hypertensive heart [38]. Taking into consideration the interplay of multiple phosphosites across regulatory myofilament proteins, cMyBP-C and cTnI phosphorylation play a direct but complex role in the regulation of Ca^2+^-dependent force production during the progression of HF.

Increased cardiomyocyte passive stiffness is also a hallmark of diastolic dysfunction [13]. The increased stiffness is independent of sex and HF phenotype as it occurs in human HFpEF [45] and HFrEF [46]. Titin phosphorylation status itself is a major contributor to cardiomyocyte passive stiffness [18], in the failing human heart the balance of titin phosphorylation by different protein kinases especially CaMKII and PKG is deranged [18]. PKG-mediated hypophosphorylation and CaMKII-mediated hyperphosphorylation of titin has been observed in HF [46, 47]. In accordance, here we found PKG-mediated hypophosphorylation, which was associated with the reduction of PKG expression and activity in TG rats of both sexes. However, this phenomenon was more prominent in females and in vitro PKG incubation corrected titin-based passive stiffness nearly completely in females, but only incompletely in males. Related to endothelial dysfunction seen in TG animals, comprehensive data support the concept of ROS-induced downregulation of the NO-soluble guanylyl cyclase (sGC)-cGMP-PKG signaling pathway leading to elevated diastolic stiffness in human HF [48]. In our previous work, we observed that LV oxidative stress in TG animals was associated with an increase in proinflammatory cytokines, as well as significant alterations in apoptotic and autophagy pathways, regardless of sex [24]. Here we also demonstrated increased PKG oxidation in the failing hearts. These results are in accordance with our former observations in human and experimental HFpEF that oxidative stress induces PKG1α oxidation, leading to the production of PKG1α dimers/polymers that are localized to the outer membrane of the cardiomyocyte and reduce PKG activity [49].

We observed correction of passive stiffness upon in vitro treatment with CaMKIIδ, but it was less pronounced in both sexes and CaMKIIδ was altered differently in TG females vs. males. CaMKIIδ action is complex. On the one hand, CaMKII directly affects titin function phosphorylating different residues, thereby regulating diastolic stress in human HF [47]. On the other hand, CaMKII is an integrative sensor of oxidative stress and Ca^2+^ signals in the heart [50]. CaMKII is activated primarily by binding of Ca^2+^-calmodulin triggering autophosphorylation at Thr287, thereby promoting full CaMKII activation [17]. Here we detected that CaMKII autophosphorylation was not altered significantly in the heart of TG or WT animals, but CaMKII activity was clearly enhanced in TG female animals. This increased activity is probably due to the increased oxidation of the enzyme in TG females. It has been described that angiotensin II-induced oxidation of paired regulatory domain Met residues also activates CaMKII in the absence of Ca^2+^/calmodulin, leading to impaired cardiac function and increased mortality after myocardial infarction [51].

In conclusion, this unique animal model enabled us to study the combined effects of age, sex and comorbidity leading to HF. In accordance with human clinical data, here we provided further mechanistic evidence of sex-specific cardiovascular remodeling in the development of HF. In our HF model, important sex differences appeared in endothelial and protein kinase-mediated cardiomyocyte dysfunction, contributing to different survival and development of HF among males and females. We believe our data serve as another step on the way to tailored HF therapy.

### Study limitation

We need to emphasize that here we used inbreeding to establish a close enough WT control group. It might indicate that it is not the transgene that is the only difference in our study population. There is another inbred property dominantly affecting sympathetic activation, especially in males, which surfaced in the study. Accordingly, WT males vs. females appeared with highly phosphorylated myofilament proteins but lessened protein kinase activities at baseline, while pCa_50_ and F_max_ were lower, and AMPK was expressed and activated more. These observations are important because little is known about an authentic control strain since conventionally [12] either SD or Lewis rats have been used for a control group comparing TG mRen2 rats with an unrelated background. Further, more detailed studies are needed to investigate sex differences in cardiac aging and sarcomeric function [52].

In addition, diastolic dysfunction in the mRen2 strain is caused by multiple factors including LV hypertrophy, fibrosis and post-translational modifications of sarcomeric proteins as previously reported [13, 24]. In cardiac diseases with energy depletion such as HF, it is also likely that elevations of intracellular ADP levels contribute to diastolic dysfunction, even at low Ca^2+^ levels [53]. Nonetheless, further studies are needed as these aspects are outside the scope of the recent work.
